# Tunable ultrasensitivity: functional decoupling and biological insights

**DOI:** 10.1038/srep20345

**Published:** 2016-02-05

**Authors:** Guanyu Wang, Mengshi Zhang

**Affiliations:** 1 Department of Biology, South University of Science and Technology of China, Shenzhen, Guangdong 518055, China

## Abstract

Sensitivity has become a basic concept in biology, but much less is known about its tuning, probably because allosteric cooperativity, the best known mechanism of sensitivity, is determined by rigid conformations of interacting molecules and is thus difficult to tune. Reversible covalent modification (RCM), owing to its systems-level ingenuity, can generate concentration based, tunable sensitivity. Using a mathematical model of regulated RCM, we find sensitivity tuning can be decomposed into two orthogonal modes, which provide great insights into vital biological processes such as tissue development and cell cycle progression. We find that decoupling of the two modes of sensitivity tuning is critical to fidelity of cell fate decision; the decoupling is thus important in development. The decomposition also allows us to solve the ‘wasteful degradation conundrum’ in budding yeast cell cycle checkpoint, which further leads to discovery of a subtle but essential difference between positive feedback and double negative feedback. The latter guarantees revocability of stress-induced cell cycle arrest; while the former does not. By studying concentration conditions in the system, we extend applicability of ultrasensitivity and explain the ubiquity of reversible covalent modification.

Sensitivity is important in biology—a cell often needs to make clear-cut decisions such as whether to commit apoptosis or which cell type to become. The best known mechanism of sensitivity is cooperative binding of substrate with enzyme that has more than one binding site. The binding of one substrate molecule may cause some allosteric change that makes the second molecule easier to bind. Zero-order ultrasensitivity, discovered by Goldbeter and Koshland^1^, is another important mechanism of sensitivity. When a substrate is under reversible covalent modification (RCM) by two opposing enzymes, the substrate state can be highly sensitive to small changes of enzyme concentration. In the following, ‘zero-order ultrasensitivity’ is often abbreviated to ‘ultrasensitivity,’ for simplicity. Because ultrasensitivity and cooperative sensitivity have no differences in appearance (both take the same sigmoidal shape), one may wonder the *functional* difference between the two.

We propose that tunability represents one major difference. Cooperative sensitivity is conformation based and is thus difficult to tune. Its Hill coefficient has a theoretical value equaling the number of binding sites^2^, which is fixed. This conformational restriction also implies low degree of sensitivity—the number of binding sites is usually small; and factors such as incomplete cooperation further reduces sensitivity. For example, hemoglobin has four binding sites for oxygen; but the actual value of the Hill coefficient is only approximately three. Ultrasensitivity, arising from the delicate balance among molecule species of a system of RCM, is concentration based. Because concentrations can be easily altered by transcriptional regulations or protein-protein interactions, ultrasensitivity should be highly tunable. Therefore, RCM may provide a wide range of sensitivity patterns and allow for plasticity in variable environments.

Using a mathematical model of regulated RCM, we find that ultrasensitivity is indeed tunable and that sensitivity tuning can be decomposed into two orthogonal modes. Under some ideal conditions that are approachable, the two kinds of tuning are decoupled (realized by crosstalk-free regulations), which confers a remarkable functional separation. These discoveries provide valuable insights into vital biological processes such as tissue development and cell cycle. In particular, we resolve a conundrum in budding yeast cell cycle checkpoint, which leads to the further discovery of a subtle but essential difference between positive feedback and double negative feedback. The latter guarantees revocability of stress-induced cell cycle arrest; while the former does not. We also find that decoupling of the two modes of sensitivity tuning is critical to fidelity of cell fate decision and is thus important in tissue development.

The advantages of ultrasensitivity highlight RCM and may explain its ubiquity. Indeed, covalent modification has many kinds (phosphorylation, methylation, acetylation, etc.) and they are all reversible. Even the cycle of phosphorylation and dephosphorylation alone is a subject of intensive studies. As of December 2015, the MEDLINE database returns over 252,000 articles upon searching ‘phosphorylation.’

It is generally regarded that ultrasensitivity depends on a condition known as enzyme saturation^1^. Because enzyme saturation is not guaranteed in some RCM systems such as some protein interaction networks, practicality of ultrasensitivity is often questioned^3^. In this paper, we find that enzyme saturation is not critical to ultrasensitivity and its fine tuning, which extends applicability of ultrasensitivity and explains the ubiquity of RCM more convincingly.

Our insights arise from decomposition of sensitivity tuning. Decomposition is very effective in clarifying complex problems. Prominent examples of decomposition include the historical decomposition of force and recent decomposition of dynamics into potential and flux components^4^. More deeply, It is idealization that leads to the decomposition, in a similar manner as the abstraction of point mass can facilitate the decomposition of force.

## Method

**Figure 1:**
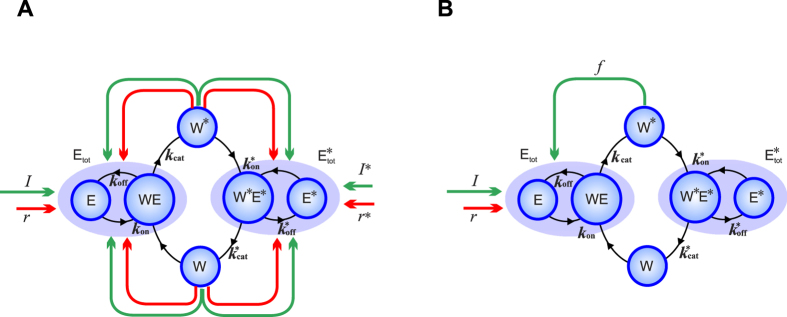
Reversible covalent modification under regulations. The transitions between the modified (W*) and demodified (*W*) substrates are controlled by two opposing enzymes *E*_tot_ and 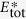, which are in turn subject to the feedback regulations from the substrates. The enzyme *E*_tot_ is divided into the free enzyme *E* and the bound enzyme *WE* to better illustrate the Michaelis-Menten kinetics. (**A**) The model with a relatively complete set of regulations. (**B**) The primary model in the present study.

Figure 1A illustrates our model system, where the blue component is the RCM originally studied in^1^: *W* and W* are the unmodified and modified substrate, respectively; *E*_tot_ and 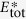 are the modifying and demodifying enzymes, respectively; *I* is some distinguished stimulus. The stimulus-response curve is represented by the function W*(*I*). Peripheral to the blue component are regulations to the enzymes, where green (red) arrows represent stimulations (inhibitions). The regulations can also be classified according to their sources: those emanated from *W* or W* are feedbacks and the others (r, I*, and r*) are nonfeedbacks.

Since RCM examples typically involve one feedback regulation^5^,^6^,^7^,^8^,^9^,^10^, We primarily study the RCM with the positive feedback from W* to *E*_tot_ (Fig. 1B). The corresponding mathematical model (Eqs (1, 2, 3, 4, 5, 6, 7, 8, 9, 10)) is called the full model.

### Full model (with the positive feedback)

The time evolution of the system is described by:


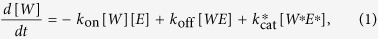



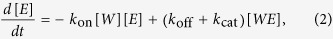



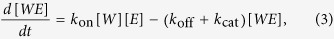



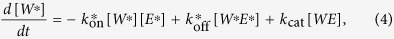



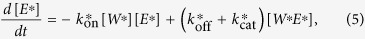



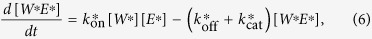



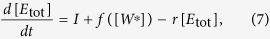



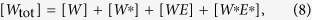



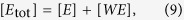



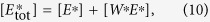


where

[⋅] represents concentration. It will often be omitted to reduce clutter.

*W*_tot_ is the total substrate, consisting of the unmodified substrate *W*, the modified substrate W*, the compounds *WE* and W*E* (Eq. (8)).

*E*_tot_ is the enzyme catalyzing *W* → W*, consisting of the free enzyme *E* and the compound *WE* (Eq. (9)). It is subject to regulation (Eq. (7)).


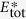
 is the enzyme catalyzing W* → *W*, consisting of the free enzyme E* and the compound W*E* (Eq. (10)).

*k*_on_ is the association constant, namely the rate of associating *W* with *E* to form the compound *WE*.

*k*_off_ is the disassociation constant, namely the rate of disassociating *W* from the compound *WE*.

*k*_cat_ is the production constant, namely the catalytic rate of producing the modified substrate W* and regenerating the free enzyme *E*.

*r* is the rate of decay of the enzyme *E*_tot_, caused by external inhibition or self-degradation.


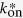
, 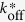, 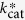, and r* are the counter-parts of *k*_on_, *k*_off_, *k*_cat_, and *r*, respectively.

*f* (W*) is the feedback from W* to *E*_tot_. The feedback is a positive one because of the plus sign before *f* (W*) in Eq. (7). See below for its expression.

We also define


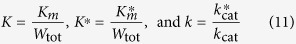


for later uses, where *K*_*m*_ = (*k*_off_ + *k*_cat_)/*k*_on_ and 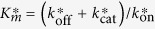 are the well-known Michaelis constants.

In the absence of feedback regulation (Eq. (7)), the mathematical model reduces to the original one studied in^1^.

### Feedbacks

#### Models of feedback (nonlinear vs. linear)

In the full model, we have used *f* (W*) to denote the feedback. As a generic assumption in biochemistry, we let it be a nonlinear, Hill function:


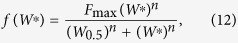


where *F*_max_ is the maximal strength, *W*_0.5_ is half maximal effective concentration of W*, and *n* is the Hill coefficient.

For simplicity, we also use the linear function


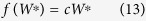


to model the feedback, at times appropriate.

#### Kinds of feedback (positive, negative, etc.)

The full model involves only with the positive feedback from W* to *E*_tot_, which is just one of the eight feedback loops illustrated in Fig. 1A. The other seven feedbacks are respectively studied, still based on Eqs (1, 2, 3, 4, 5, 6, 7, 8, 9, 10) but with Eq. (7) replaced. See Fig. S.3 for the eight feedback models (column A) and their replaced equations (column B). In the replaced equations, the sign before feedback *f* (⋅) indicates the kind of feedback. The following are three examples.

In Fig. S.3(B1), the term +*f* (W*) implies that the feedback is positive, which conforms with the green arrow *f* in Fig. S.3(A1).

In Fig. S.3(B2), the term −*f* (W*)*E*_tot_ implies that the feedback is negative, which conforms with the red arrow *f* in Fig. S.3(A2).

In Fig. S.3(B4), the term −*f* (*W*)*E*_tot_ implies that the feedback is negative. This feedback emanates from *W*, which is in some sense ‘negative’ to W* (the increase of *W* corresponds to the decrease of W*). Therefore, this feedback can be termed *double negative feedback* from the perspective of W*. In fact, the term has been used by King *et al.* to describe the feedback regulation of the RCM of the protein complex Clb/Cdc28^11^.

### Steady states

To obtain the steady states, the left hand sides of Eqs (1, 2, 3, 4, 5, 6, 7) are first replaced with zero, which results in ten algebraic equations in total. Given a stimulus *I*, the ten equations are solved numerically to obtain the steady state values, including W*. By sweeping *I* from small to large, the response curve W*(*I*) is obtained. The curve is either bistable (Fig. S.1*A*) or graded (Fig. S.1*B*).

A bistable curve can be divided into three branches (lower, middle, upper) according to the two thresholds *I*_on_ (the activation threshold) and *I*_off_ (the deactivation threshold). When the stimulus is smaller than *I*_off_, nearly no substrates are modified; when the stimulus is greater than *I*_on_, nearly all substrates are modified; when the stimulus is between *I*_off_ and *I*_on_, the response adheres to its current state, which is either modification or demodification. We define Δ*I* = *I*_on_ − *I*_off_ and call it the hysteresis width.

### Stability analysis

The middle branch of a bistable response is indicated by dashed curve (Fig. S.1*A*), because it is generally assumed unstable and is thus absent in reality. That is why the response is called bistable instead of tristable. For assurance, we perform stability analysis on about 6000 response curves (see below), according to the method described in Supplementary Information. As expected, a point is always unstable if it belongs to the middle branch of a bistable curve; it is always stable for all the other cases (the graded response, the upper branch bistable response, and the lower branch bistable response).

### Bifurcation analysis

If the aim is to obtain *I*_on_ and *I*_off_ only, one needs not to trace out the entire response curve as described above. Given that *I*_on_ and *I*_off_ are bifurcation points, they can be obtained by a one-time solution of a set of algebraic equations (characterizing both steady state and bifurcation conditions). See Supplementary Information for details and^12^ for further information. A byproduct of the method is quick determination of the type of the response curve. If the equations have reasonable solutions to *I*_on_ and *I*_off_, then the response is bistable. Otherwise the response is graded.

### Idealized model

By using ideal conditions *K* = 0, K* = 0, and *WE* + W*E* = 0 (see Supplementary Information), a closed-form solution


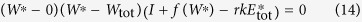


is obtained. The three factors in the solution correspond to the three branches of a bistable switch: lower, upper, and middle (Fig. S.2*A*), which endows intuitive geometric meaning to *I*_on_ and *I*_off_: the point (*I*_on_, 0) is the intersection of the middle and the lower branches; while the point (*I*_off_, *W*_tot_) is the intersection of the middle and the upper branches. We thus obtain the closed-form expressions of *I*_on_ and *I*_off_ (Eq. (S.41)). By using Δ*I* = *I*_on_ − *I*_off_ to replace *I*_off_, we obtain


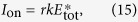



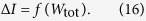


The above elucidation is also applicable when the feedback is linear (Fig. S.2*D* – *F*).

### Validation of the idealized model

We validate idealization by producing full model responses with small *K* and K* values (Fig. S.1*A*). These responses are indeed close to the idealized model response (Fig. S.2*A*). In particular, the green curve has *I*_on_ = 0.0448 and Δ*I* = 0.0392, which are very close to the idealized model results *I*_on_ = 0.045 and Δ*I* = 0.0397 (obtained by Eqs. (15 and 16)).

**Figure 2:**
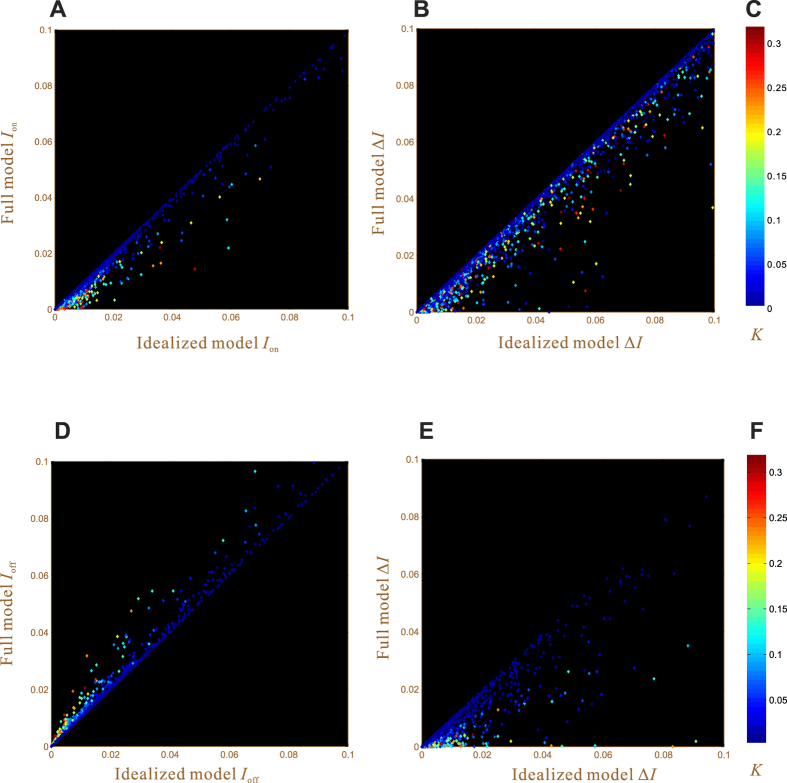
Comparison of the idealized model results (the horizontal axis) and the full model results (the vertical axis). The upper panels are for the positive feedback model. The lower panels are for the double negative feedback model. The feedbacks are in the form of the Hill function. Each dot corresponds to a randomly generated response curve. The random parameters used to produce the response curves are within the following ranges: *W*_tot_ = 1, 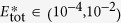, *K* ∈ (10^−4^, 10^−0.5^), K* = *K, r* ∈ (1, 2), *W*_0.5_ ∈ (0.2, 1), *n* ∈ (2, 5), *F*_max_ ∈ (10^−3^, 10^−0.5^) (for the upper panels), *F*_max_ ∈ (10^−1^, 10^−0.5^) (for the lower panels). The color of each dot encodes its *K* value. (**A**) The *I*_on_ value. (**B**) The Δ*I* value. (**C**) The color bar for *K*. (**D**) The *I*_off_ value. (**E**) The Δ*I* value. (**F**) The color bar for *K*.

Bifurcation analysis allows for the direct calculation of (*I*_on_, Δ*I*) of the full model response, without tracing out the response curve. We randomly generate about 6000 sets of parameter values and calculate their respective (*I*_on_, Δ*I*). We find that about 1000 cases do not have reasonable solutions; they correspond to graded curves and are thus excluded from the present study. The remaining 5000 cases all have reasonable solutions, which are presented as 5000 dots in Fig. 2A. For a given dot, its vertical coordinate represents the full model *I*_on_ determined by bifurcation analysis; its horizontal coordinate represents the idealized model *I*_on_ determined by Eq. (15); its color encodes the value of *K*. Most of the dots accumulate around the diagonal, demonstrating that Eq. (15) gives good approximation for *I*_on_. Figure 2B presents the comparison of Δ*I*, which has the same pattern as Fig. 2A but with more scattered dots; thus the approximation of Δ*I* is less accurate. Another observation is that the dots are always subdiagonal, indicating that the full model *I*_on_ and Δ*I* are always smaller than the idealized model counterparts. This observation actually conforms with Fig. S.1*A*, which shows that the full model response curves are all enveloped within the idealized model curve.

## Results and Discussions

### Decomposition of sensitivity tuning

**Figure 3:**
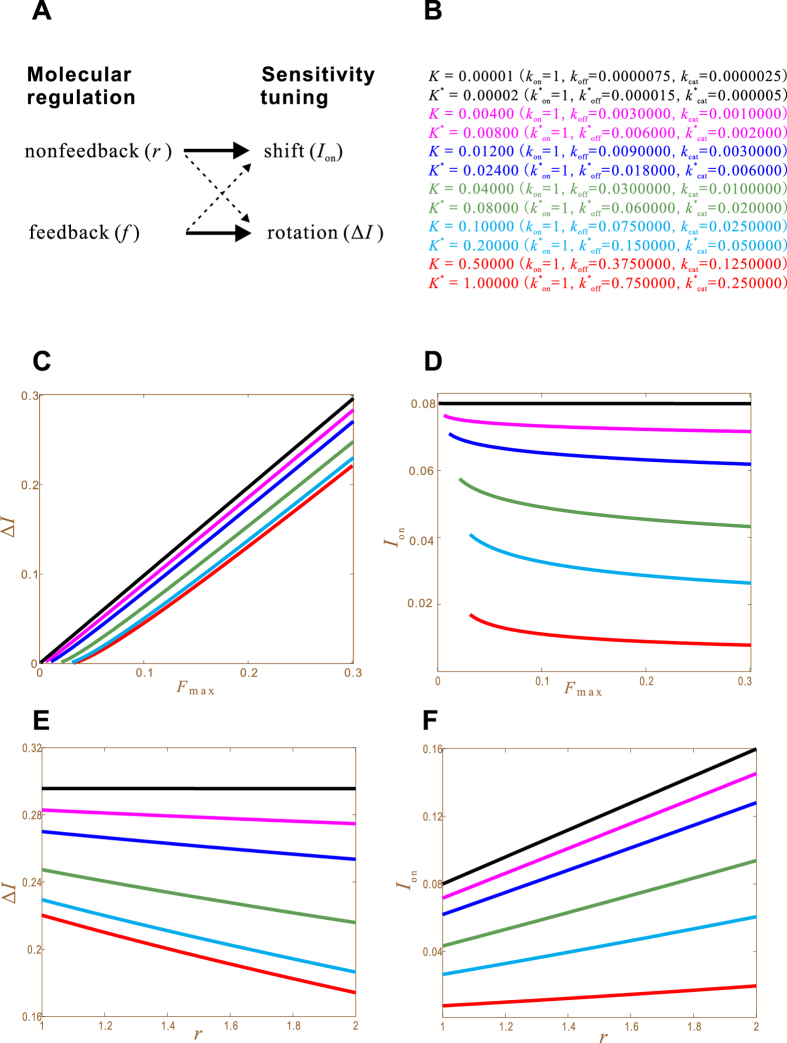
Causal relationships between molecular regulations (represented by *F*_max_ and *r*) and sensitivity tuning (changes of *I*_on_ and Δ*I*). The common parameter values are *W*_tot_ = 1, 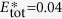, *W*_0.5_ = 0.3, *n* = 4, *r* = 1 (when *F*_max_ is varied), *F*_max_ = 0.04 (when *r* is varied). (**A**) Targeted regulations (solid arrows) and crosstalks (dashed arrows). (**B**) Six sets of *K* and K* values. (**C**) Δ*I* as a function of *F*_max_. Different curves correspond (through color) to different *K* and K* values. (**D**) *I*_on_ as a function of *F*_max_. (**E**) Δ*I* as a function of *r*. (**F**) *I*_on_ as a function of *r*.

Equations (15 and 16) demonstrate a one-to-one correspondence between kinds of molecular regulation and kinds of sensitivity tuning: the nonfeedback *r* regulates the activation threshold *I*_on_; the feedback *f* regulates the hysteresis width Δ*I*. Under ideal conditions, there are no crosstalks: *r* does not affect Δ*I* and *f* does not affect *I*_on_ (Fig. 3A).

#### The first kind of sensitivity tuning: SHIFT

**Figure 4:**
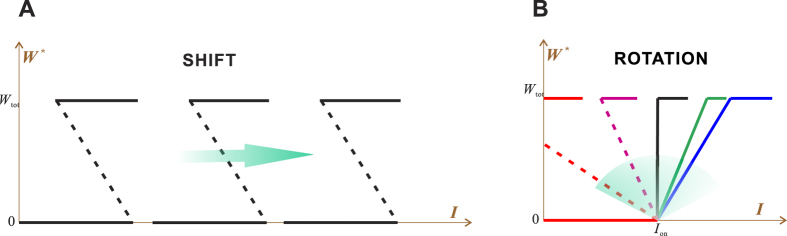
Sensitivity tuning under the ideal conditions and with the linear feedback. (**A**) The first kind: SHIFT. (**B**) The second kind: ROTATION.

Under ideal conditions, the nonfeedback regulation *r* determines the activation threshold and thus timing of sensitivity onset (Eq. (15)). If *r* is an inhibition as illustrated in Fig. 1, then as *r* increases, the response curve shifts to the right, reducing the chance of activation (Fig. 4A). But once activated, the response is still maximal despite the inhibition, because the inhibition only shifts the response curve but does not distort it.

#### The second kind of sensitivity tuning: ROTATION

Under ideal conditions, the positive feedback simply generates the hysteresis width (Eq. (16)). We now consider the negative feedback from W* to *E*_tot_ and obtain the corresponding idealized model, from which *I*_on_ and Δ*I* are obtained (Fig. S.3(C2)). While *I*_on_ remains the same as in the positive feedback, Δ*I* turns from +*f* (*W*_tot_) to 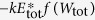, which corresponds to flipping the middle branch from the left side of *I*_on_ to the right, with possibly some skewness because 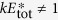 in general (Fig. S.2*B*). Figure S.2*C* displays response curve of positive (blue, green), negative (red, magenta), and null (black) feedbacks. As the feedback changes, the response curve ‘rotates’ around the fixed *I*_on_.

By using the linear feedback *f* (W*) = cW*, the middle branch becomes a straight line, which makes the rotation more intuitive (Fig. 4B). It provides an intuitive explanation of a cell’s *sensitive robustness*^13^, a seemingly self-contradictory property. It is well-known that positive feedback can augment sensitivity. Consider the blue lines in Fig. 4B, which represent a low sensitivity response. To enhance the sensitivity, we apply a positive feedback. As the feedback strength increases, the middle branch rotates counterclockwise and becomes vertical (the maximal sensitivity). Further increase of the feedback strength won’t increase sensitivity; but the rotation continues to generate hysteresis, which confers robustness. Without hysteresis, the achieved high sensitivity would have an undesired side effect, namely fragility. For example, the stimulus *I* may fluctuate around *I*_on_, switching the substrate incessantly between the unmodified and modified states (the clattering phenomenon). It is the hysteresis width Δ*I* that buffers the undesired switching and stabilizes the modification. Therefore, the same positive feedback achieves both sensitivity augment and robustness generation, unifying the two opposing properties that are both important in biology.

As the feedback becomes even stronger, the left corner of the response curve is cut off by the vertical axis (i.e., *I*_off_ becomes negative, see the red lines in Fig. 4B), which produces a ‘ratchet effect’^14^. As long as the stimulus *I once* exceeds the threshold *I*_on_ and causes modification, the modification pertains even after a complete withdrawal of the stimulus (*I* reducing to 0), producing a memory of the transient stimulus, or irreversibility^15^. The irreversibility is of central importance in certain biological processes such as the one-way progression of cell cycle G1 → S → G2 → M → G1, where absolute irreversibility is required^16^.

#### Crosstalks

The complete decoupling of sensitivity tuning occurs when *K* and K* are zero. As they deviate from zero, crosstalks cartainly emerge. We test this by using *K* and K* values listed in Fig. 3B. When they are on the order of 10^−5^, the crosstalks are negligible: *F*_max_ does not affect *I*_on_ (the black line in Fig. 3D) and *r* does not affect Δ*I* (the black line in Fig. 3E); while the targeted regulations *F*_max_ → Δ*I* and *r* → *I*_on_ are linear functions (the black lines in Fig. 3C,F, respectively). As *K* and K* increase, the crosstalks increase and the targeted regulations degrade. For the case *K* = 0.5, the crosstalk *r* → Δ*I* is even greater than the targeted regulation *r* → *I*_on_. Indeed, the slope of the red line in Fig. 3E (about 0.046) is larger than the slope of the red line in Fig. 3F (about 0.012). These results demonstrate that the decoupling of SHIFT and ROTATION depends on the smallness of *K* and K*.

### Concentration conditions for ultrasensitivity

Because ultrasensitivity is concentration based, it is of central importance to clarify general concentration conditions that critically affect ultrasensitivity. Enzyme saturation has long been regarded as a necessary condition for ultrasensitivity—the widespread term ‘zero-order ultrasensitivity’ just means ‘ultrasensitivity arising from enzyme saturation.’ Because enzyme saturation does not always hold (e.g., some protein interaction networks have enzymes and substrate concentrations at the same order of magnitude), practicality of ultrasensitivity is often questioned^3^,^17^. Here we emphasize that it is ‘substrate abundance,’ a condition closely related to enzyme saturation, that is truly necessary for ultrasensitivity.

#### Substrate abundance is necessary for ultrasensitivity

The smallness of *K* and K* is criticial to ultrasensitivity and its fine tuning. First, it determines the degree of sensitivity. The smaller *K* and K*, the sharper the switching behavior (Fig. 1 of ref. 1). Second, it confers the decoupling of sensitivity tuning.

According to Eq. (11), *K* and K* are Michaelis constants divided by *W*_tot_. Because the Michaelis constants are relatively invariant, the smallness of *K* and K* actually depends on the largeness of *W*_tot_, namely substrate abundance.

#### Substrate abundance does not always lead to enzyme saturation

Enzyme saturation is a quite common condition. Because an enzyme molecule can be repetitively used, the copy number of an enzyme needs not to be large. Therefore, an enzyme molecule is usually saturated with substrates, especially when substrates are abundant. In metabolic systems, for example, metabolite (substrate) concentrations are orders of magnitude larger than enzyme concentrations^3^. But substrate abundance does not always lead to enzyme saturation. It is possible that substrate and enzyme are in comparably high concentrations, a case of substrate abundance, enzyme abundance, but not enzyme saturation.

#### Enzyme saturation enhances ultrasensitivity but is not absolutely required

The difference between substrate abundance and enzyme saturation makes it necessary to investigate the latter more carefully. We use the ratio 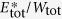 to measure enzyme saturation, which is equal to 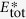 because of the normalization *W*_tot_ = 1. The smaller 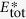 is, the enzyme is more saturated. For example, 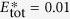 is certainly a case of saturation—the substrate is 100 folds as many as the enzyme; 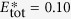 is a marginal case; 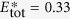 is a case of unsaturation—an enzyme is surrounded by only three substrates in average. Before the investigation, it should first be noted that 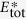 itself is a nonfeedback regulation, which confers the same SHIFT as *r* does (Eq. (15)). Therefore, the increase of 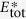 has both the regulatory function and the adverse effect of making the enzyme less saturated.

**Figure 5:**
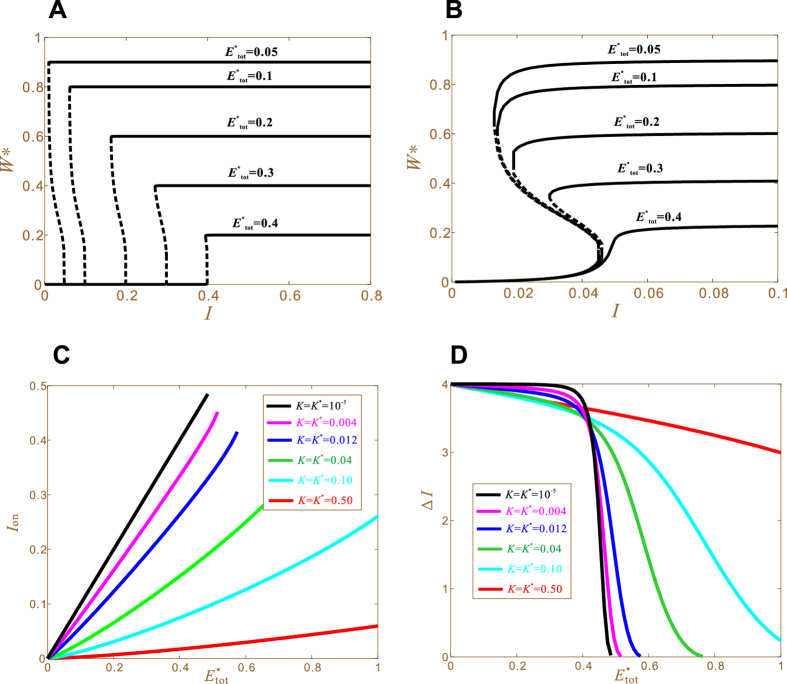
Effects of the enzyme concentration 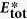 on sensitivity. (**A**) Five response curves W*(*I*) that are obtained with five 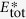 values and the same other parameters: *K* = K* = 10^−5^, *W*_tot_ = 1, *r* = 1, *F*_max_ = 0.04, *W*_0.5_ = 0.3, *n* = 4. (**B**) Five response curves obtained under the same conditions as (A) except the following two differences. First, *K* = K* = 10^−2^. Second, the curves are different in both 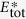 and *r* because we have fixed 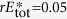. (**C**) Effects of 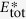 on *I*_on_ obtained with six sets of *K* and K* values. The other parameters are the same as in (**A**). (**D**) Effects of 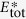 on the hysteresis width Δ*I*.

Figure 5A demonstrates consequences of increasing 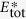 for the case *K* = K* = 10^−5^. First, the response curve shifts to the right, manifesting the regulatory function (Eq. (15)). Second, the maximal response W* reduces, because the increase of 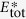 renders the increase of *WE* + W*E* and the consequential reduction of *W* + W* (Eq. (8)). Finally, all the curves maintain sharp sensitivity, even for cases of enzyme unsaturation (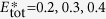). Figure 5B is for the case *K* = K* = 0.01. This time we fix 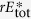 to make the theoretical value of *I*_on_ the same for all the curves; thus the increase of 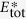 is always accompanied by the corresponding decrease of *r*. As 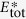 increases, sensitivity degrades only mildly: all the curves keep good sigmoidal shape; the *I*_on_ value only changes slightly; the Δ*I* value is relatively more affected. On the other hand, the maximal response reduces markedly, as expected. These results again demonstrate that substrate abundance is the determinant of ultrasensitivity. As long as *K* and K* are sufficiently small, ultrasensitivity is well maintained even if enzymes are not saturated.

We then study the effects of 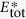 on *I*_on_. Because 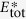 and *r* affect *I*_on_ equally under ideal conditions (Eq. (15)), we expect that the functions 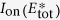 and *I*_on_(*r*) should be similar, which is indeed the case (cf. Figs 3F and 5C). Their deviation is an effect of enzyme unsaturation. Because the deviation is small, enzyme saturation is not critical to the tuning of *I*_on_. In fact, Fig. 5A,B have already demonstrated immunity of *I*_on_-tunability from the state of saturation.

We finally study the effects of varying 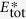 on Δ*I* (Fig. 5D). When 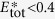, Δ*I* changes little as 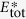 varies. This conforms with the ideal condition result that Δ*I* is independent of 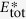 (Eq. (16)). Even for the worst case *K* = K* = 0.1, Δ*I* only reduces about 10% when 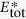 reaches 0.4. When 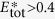, the invariance of Δ*I* fails completely; Δ*I* reduces dramatically as 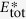 increases. Therefore, although enzyme saturation is not absolutely required, the enzyme concentrations should not be exceedingly large; they should always keep less than 40% of the substrate concentration.

#### Practicality of ultrasensitivity

Our results have demonstrated that substrate abundance, but not enzyme saturation, is the determinant of ultrasensitivity. Although the increase of enzyme concentration certainly degrades sensitivity, the ill-effects are still tolerable even when apparent enzyme unsaturation is reached, e.g., one enzyme is surrounded by only three substrates (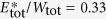). Therefore, ultrasensitivity may exist in RCM systems that have comparable magnitudes of enzyme and substrate, such as some protein interaction networks. In such systems, the maximal output is significantly less than *W*_tot_, but the switching between minimum and maximum is still sensitive. The results thus extend the applicability of ultrasensitivity and more convincingly explain the ubiquity of reversible covalent modification. Of course, the extension is not without limitations. Figure 5D has demonstrated that the enzymes must be less than at least 40% of the substrates.

### Insights into cell cycle checkpoint

#### The wasteful degradation conundrum

**Figure 6:**
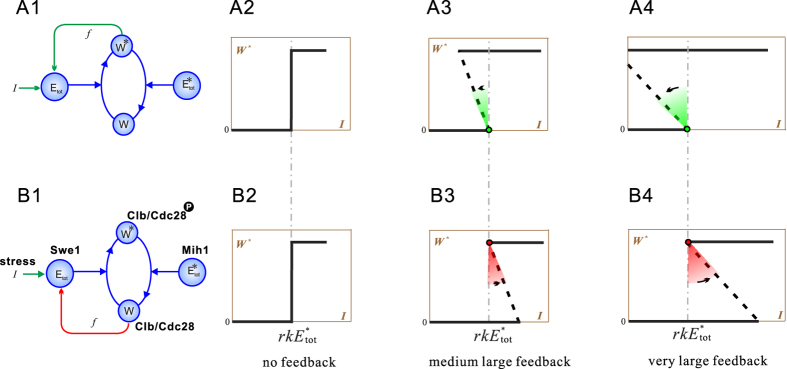
Comparison of the positive feedback model (upper) and the double negative feedback model (lower). The first column presents the RCM model. The second, third, and fourth columns present response curves with null, small, and large feedbacks.

The transition of budding yeast cell cycle from the G2 phase to the M phase depends on the formation and activation of the complex Clb/Cdc28. In response to stresses such as hyperosmotic shock and nutrient depletion, the protein kinase Swe1 establishes, which phosphorylates and then inactivates Clb/Cdc28, leading to cell cycle arrest until the removal of the stress^18^,^19^. This checkpoint system is represented by the RCM in Fig. 6(B1), where I, W, W*, *E*_tot_, and 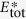 correspond to the stress, Clb/Cdc28, phospho-Clb/Cdc28, Swe1, and Mih1, respectively; the inhibitory edge from *W* to *E*_tot_ corresponds to the fact that Clb/Cdc28 inhibits Swe1 and leads to its degradation. Because *W* is in some sense ‘negative’ to W*, the inhibitory edge from *W* to *E*_tot_ was regarded as a double negative feedback from W*^11^, whose effects should be similar to the positive feedback from W* to *E*_tot_.

The double negative feedback involves repetitive and massive synthesis and degradation of Swe1 and seems wasteful. Before the formation of Clb/Cdc28, Swe1 has reached a large concentration, owing to its consistent synthesis throughout late G1, S, and early G2 phases^19^,^20^. The accumulated Swe1 is rapidly degraded upon the formation of Clb/Cdc28 (because Clb/Cdc28 inhibits Swe1), leaving a trace level of Swe1 that is so maintained until the *next* cycle, when Swe1 synthesis begins again. This synthesis-accumulation-degradation activity of Swe1, which repeats for every cell cycle, is very expensive, because protein synthesis and degradation are energy costly.

Why didn’t nature use the positive feedback from W* to *E*_tot_ (Fig. 6(A1)), which is more direct and economic? If the positive feedback is used, Swe1 needs only to maintain at a low concentration under normal conditions. A high Swe1 concentration establishes only through the induction by stress and the reinforcement by the positive feedback (*I* → *E*_tot_ → W* → *E*_tot_). In this way, the positive feedback guarantees that massive Swe1 synthesis occurs *only* in the presence of stress, which is certainly better than the double negative feedback from a design perspective: expensive activities operate only when necessary.

It turns out that double negative feedback has a subtle but essential difference from the positive feedback, which makes it absolutely necessary. The discovery is due to insights obtained from idealization and decomposition—the two feedbacks have different pivots of ROTATION, which consititutes the major difference between the two.

#### Difference between positive feedback and double negative feedback

Figure 6(A1) reproduces the positive feedback model. Its closed-form solution (Eq. (14)) implies the following. For a more intuitive presentation, here we use the linear feedback *f* (W*) = cW*. In the absence of feedback (*c* = 0), the middle branch is a vertical centerline whose horizontal coordinate is 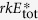 (Fig. 6(A2)). As the feedback increases (*c* > 0), the middle branch rotates around the green dot counterclockwise; thus it enters the *left* hand side of the centerline (Fig. 6(A3)). Note that the resultant bistability still has 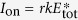.

Figure 6(B1) illustrates the double negative feedback model. Its closed-form solution (Fig. S.3(C4)) implies the following. In the absence of feedback (*c* = 0), the middle branch is a vertical centerline whose horizontal coordinate is 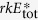 (Fig. 6(B2)). As the feedback increases (*c* > 0), the middle branch rotates around the red dot counterclockwise; thus it enters the *right* hand side of the centerline (Fig. 6(B3)). Note that the resultant bistability has 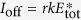 (not 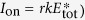.

At first glance, the two feedbacks have no essential differences, because the two bistable responses can be tuned identical by adjusting parameters. However, a fundamental difference emerges when *c* is sufficiently large. For the positive feedback, *I*_off_ becomes negative, which leads to irreversibility (Fig. 6(A4)). For the double negative feedback, *I*_off_ is fixed and always positive, which guarantees reversibility (Fig. 6(B4)). The reversibility is crucial for budding yeast to resume cell cycle as the stress fades away: once *I* drops below *I*_off_, Clb/Cdc28 dephosphorylates completely.

The invariance of *I*_off_ results from the idealized model of double negative feedback. Under practical conditions, the feedback does perturb *I*_off_ through crosstalk, which may lead to a negative *I*_off_. We thus perform statistical analysis to compare full model *I*_off_ and idealized model *I*_off_ (Fig. 2D). One sees that full model *I*_off_ are always larger than idealized model *I*_off_ (because the dots are always superdiagonal). In other words, the double negative feedback always makes the actual *I*_off_ larger than 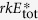, ruling out the possibility that *I*_off_ becomes negative. Therefore, reversibility of checkpoint arrest is even more guaranteed in reality.

#### Solving the conundrum

The ‘wasteful’ behavior of budding yeast now has a reasonable explanation. The cell cycle arrest should have a stable duration so that stress induced damages can be repaired on time. The required duration (hysteresis) can be supplied by both positive feedback and double negative feedback. But the positive feedback may lock the system in a permanently arrested state (even after the stress is completely removed), which is fatal to the yeast colony and should be prohibited. By using the double negative feedback, revocability of cell cycle arrest is guaranteed; but the cells have to consistently pay heavy insurance—the cycle of Swe1 synthesis, accumulation, and degradation—in preparation for various stresses that may or may not occur.

### Insights into development

**Figure 7:**
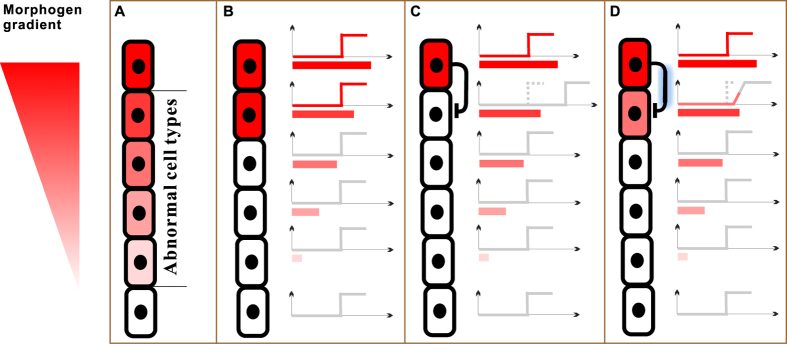
Four outcomes of cell type specification rendered by a morphogen gradient distributed over a column of six cells. (**A**) Graded response, which results in too many “ cell types.” The middle four cells may be abnormal due to partially expressed genes. (**B**) Threshold response. Only the upper two cells differentiate into a new cell type, because their local morphogen concentrations exceed *I*_on_. (**C**) Threshold response with lateral inhibition (indicated by the bar-headed arrow). Assuming the lateral inhibition is crosstalk-free. Differentiation of the trailing cell is prohibited due to the raised threshold. (**D**) Threshold response with lateral inhibition. Assuming severe crosstalk (indicated by the glowing bar-headed arrow). The trailing cell’s gene becomes partially expressed, resulting in an abnormal cell type.

Threshold response is of paramount importance in development, which involves induction of a new cell type from initially homogenous cells by a morphogen. As the morphogen diffuses from its source, a gradient is established. Responses to the morphogen gradient should be binary to elicit clear-cut cell fate specification: cells closer to the morphogen source are induced to differentiate, while the other cells should not^21^,^22^,^23^. If the response were graded, then too many ‘cell types’ would result, most of which carry aberrant gene expressions (Fig. 7A). With the binary response, a morphogen gradient specifies only two normal cell types (Fig. 7B), a desired result.

Tuning of threshold is also necessary in development. Under some circumstances (see Supplementary Information), only one cell is allowed to differentiate, which can be achieved by raising the adjacent cell’s *I*_on_ to a value unreachable by the cell’s local morphogen concentration (Fig. 7C). To that end, lateral inhibition upon the adjacent cell (indicated by the bar-headed arrow) is needed, which can be rendered by e.g. Notch/Delta signalling^24^,^25^,^26^,^27^. Note that the lateral inhibition is certainly a nonfeedback regulation, because the source is from outside of the targeted cell. It is here that the decoupling of sensitivity tuning shows its importance: it is desired that the lateral inhibition only raises the threshold but not abolishes it. Figure 7D illustrates the ill-effects of severe crosstalk (indicated by the glowing bar-headed arrow). On one hand, the targeted regulation is weakened; and the right shift becomes too small to avoid activation of the adjacent cell. On the other hand, the crosstalk renders a clockwise rotation of the response; and thus the threshold becomes obscure. The combined effect is partial activation of the adjacent cell, which may consequentially become abnormal.

Our discovery of RCM mediated functional decoupling provides a solution to the above developmental problem. By maintaining an abundant substrate pool, crosstalks of regulation becomes insignificant; and the lateral inhibition would faithfully raise the threshold without abolishing it. In Supplementary Information, we provide an example of lateral inhibition of RCM during the development of the Drosophila trachea^24^.

The possibility that RCMs define developmental thresholds was first envisioned by^28^ and later verified by experiments^29^,^30^. Melen *et al.* discovered a binary response rendered by the RCM between Yan (*W*) and phospho-Yan (W*). The response is initiated by a morphogen (*I*) such as Bnl (orthologs of mammalian fibroblast growth factor). The morphogen activates MAPK (*E*_tot_), which promotes the phosphorylation of Yan, a transcription repressor. Phospho-Yan soon degrades, which liberates multiple gene transcription and finally leads to cell differentiation. By a combination of experimental and computational studies, Melen *et al.* were able to show that only ultrasensitivity can account for the generation of threshold observed in their experiments. In Supplementary Information, we also demonstrate how intracellular nonfeedback regulations allow for the use of a single morphogen gradient to specify three or more cell types, which is a quite common situation in developmental biology^31^.

## Summary

The term ‘sensitivity’ was used in the literature with ambiguity—it refers to both switch-likeness and timing of a response. The ambiguity is exacerbated by the fact that a regulation, whether feedback or not, can affect both aspects of sensitivity. The coupling also constitutes daunting difficulties in regulating complex biological processes such as organogenesis and cell cycle progression. From a design perspective, decoupling is certainly desired to make the regulation manageable.

We found that tunable sensitivity can be rendered by the cycle of modification and demodification of a protein substrate; and that sensitivity tuning can be decomposed into two orthogonal kinds: ROTATION and SHIFT, speaking in kinematics terms. Crosstalks between the two modes can be reduced by increasing the substrate concentration, which confers, at least in principle, the wonderful property of functional separation. The decomposition has provided valuable biological insights, which allowed us to solve the wasteful degradation conundrum of budding yeast cell cycle checkpoint. We expect that decomposition of sensitivity tuning will be useful in analyzing larger networks consisting of several mutually regulated RCMs and will illuminate broader biological fields in the future.

## Additional Information

**How to cite this article**: Wang, G. and Zhang, M. Tunable ultrasensitivity: functional decoupling and biologicalinsights. *Sci. Rep.* **6**, 20345; doi: 10.1038/srep20345 (2016).

## Supplementary Material

Supplementary Information
