# Supplementary material for: Tunable ultrasensitivity: functional decoupling and biological insights
Source: Sci Rep. 2016 Feb 5;6:20345. doi: 10.1038/srep20345 (PMC4742884; doi:10.1038/srep20345)
Supplement: Supplementary Information [file srep20345-s1.pdf]

Supplementary Information

Tunable ultrasensitivity: functional decoupling and biological insights

*Guanyu Wang** and *Mengshi Zhang*

Department of Biology,

South University of Science and Technology of China,

Shenzhen, Guangdong 518055, China

*__Corresponding author: wanggy@sustc.edu.cn__

Full model of RCM with positive feedback

The mathematical model has been given by Eqs. (1–10), which describe the time evolution of the system. To obtain the steady states, the left hand side of Eqs. (1–7) are first replaced with 0, which results in ten algebraic equations in total

$$0 = -k_{\text{on}}\,[W]\,[E] + k_{\text{off}}\,[WE] + k^*_{\text{cat}}\,[W^*E^*]\,, \tag{S.1}$$

$$0 = -k_{\text{on}}\,[W]\,[E] + (k_{\text{off}} + k_{\text{cat}})\,[WE]\,, \tag{S.2}$$

$$0 = k_{\text{on}}\,[W]\,[E] - (k_{\text{off}} + k_{\text{cat}})\,[WE]\,, \tag{S.3}$$

$$0 = -k^*_{\text{on}}\,[W^*]\,[E^*] + k^*_{\text{off}}\,[W^*E^*] + k_{\text{cat}}\,[WE]\,, \tag{S.4}$$

$$0 = -k^*_{\text{on}}\,[W^*]\,[E^*] + (k^*_{\text{off}} + k^*_{\text{cat}})\,[W^*E^*]\,, \tag{S.5}$$

$$0 = k^*_{\text{on}}\,[W^*]\,[E^*] - (k^*_{\text{off}} + k^*_{\text{cat}})\,[W^*E^*]\,, \tag{S.6}$$

$$0 = I + f\,([W^*]) - r\,[E_{\text{tot}}]\,, \tag{S.7}$$

$$[W_{\text{tot}}] = [W] + [W^*] + [WE] + [W^*E^*]\,, \tag{S.8}$$

$$[E_{\text{tot}}] = [E] + [WE]\,, \tag{S.9}$$

$$[E^*_{\text{tot}}] = [E^*] + [W^*E^*]\,. \tag{S.10}$$

Response curve

Given an input I, the ten equations are solved numerically to obtain the steady state values, including W^*. By sweeping I from small to large and calculating the corresponding W^*, the response curve $W^*(I)$ is obtained. Note that a given I corresponds to multiple solutions, some of which are meaningless (e.g., $E < 0$, $W^* > W_{\text{tot}}$, etc.). Even with those meaningless solutions discarded, a given I may still correspond to several solutions.

The black response curve in Fig. S.1A gives such an example. If I is in the range $(I_{\text{off}}, I_{\text{on}})$, where $I_{\text{on}} = 0.0444$ and $I_{\text{off}} = 0.0066$, then a single I corresponds to three meaningful W^* values. The middle one of the three is always found to be unstable (it is destroyed by even the slightest

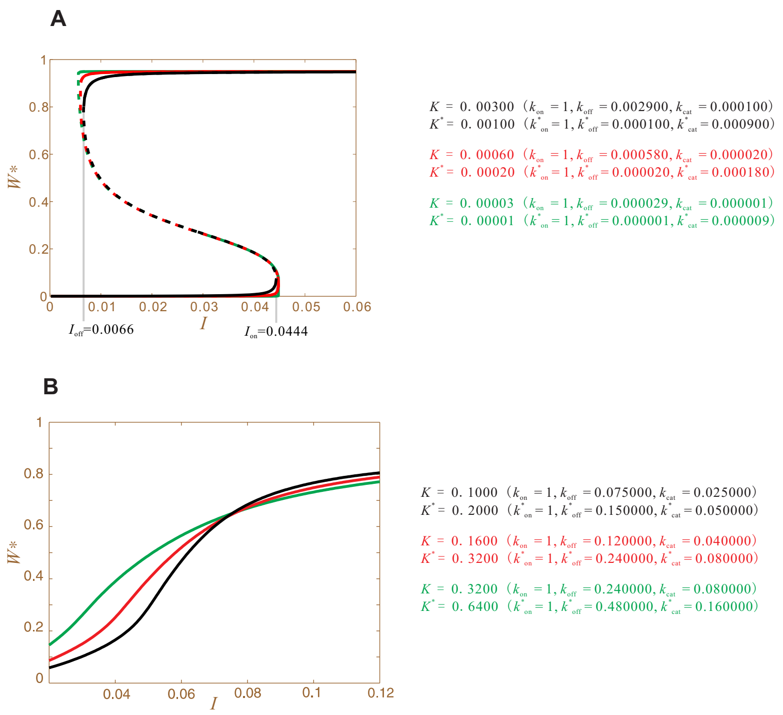

Figure S.1: **Response curves of RCM with feedback from W^* to $E_{\mathbf{tot}}$.** The feedbacks are all in the form of the Hill function. (**A**) Three bistable curves obtained from the full model. Common parameter values: $W_{\text{tot}} = 1$, $E^*_{\text{tot}} = 0.005$, $r = 1$, $F_{\max} = 0.04$, $W_{0.5} = 0.3$, $n = 4$. Curve-specific parameter values are listed besides the curves. (**B**) Three graded curves obtained from the full model. Common parameter values are the same as (A) except that $F_{\max} = 0.01$. The curve specific parameter values are listed besides the curves.

random perturbation) according to our stability analysis below. These middle values constitute the middle branch of the response curve. To signify its nonexistence in reality, the middle branch is represented by dashed curve segment. The upper and lower branches are always found to be stable. The response curve thus manifests bistability, not tristability. The red and green response curves are obtained by reducing K and K^* smaller and smaller, while keeping the other parameter values fixed. One sees that as K and K^* decrease, the response curve approaches to a limit, which will be determined by an idealized model in the following. Note that the mathematical model does not always produce bistable curves. If positive feedback is not sufficiently strong, and/or the K and K^* values are too large, graded response curves may be obtained (Fig. S.1B).

Bifurcation analysis

The numerical calculation of thresholds I_{on} and I_{off} is of paramount importance in this paper. For example, the production of Fig. 2 demands numerous calculations of I_{on} and I_{off}. If a pair of I_{on} and I_{off} can be determined only after tracing out the entire response curve (as described above), then the computational costs would be too high. Indeed, if a response curve consists of 1000 points, then the set of 10 equations (Eqs. (S.1–S.9)) have to be solved 1000 times just to plot the response curve. Additional algorithms are then required to determine I_{on} and I_{off}.

Fortunately, singularity and bifurcation theory offers an efficient method, which involves solving a set of 10 equations (Eqs. (S.13–S.22), see below) only *once* for a single pair of I_{on} and I_{off}. Let W^*_{on} and W^*_{off} denote the vertical coordinates corresponding to I_{on} and I_{off}, respectively. According to singularity theory [12], the two points (I_{off}, W^*_{off}) and (I_{on}, W^*_{on}) are singularities of the type known as *limit point*, which satisfy the following normal form equations

$$G = 0 \tag{S.11}$$

$$\partial G/\partial W^* = 0 \tag{S.12}$$

where G is the combined steady state equation (Eqs. (S.1–S.10) lumped together). The expansion

of Eqs. (S.11 and S.12) leads to the following equations:

$$(K_m + W)\,[WE] - W \cdot E_{\rm tot} = 0 \tag{S.13}$$

$$(K_m^* + W^*)\,[W^*E^*] - W^* \cdot E_{\rm tot}^* = 0 \tag{S.14}$$

$$k_{cat}^*[W^*E^*] - k_{cat}[WE] = 0 \tag{S.15}$$

$$I + f\,(W^*) - rE_{\rm tot} = 0 \tag{S.16}$$

$$W + W^* + [WE] + [W^*E^*] - W_{\rm tot} = 0 \tag{S.17}$$

$$\partial\left((K_m + W)\,[WE] - W \cdot E_{\rm tot}\right)/\partial W^* = 0 \tag{S.18}$$

$$\partial\left((K_m^* + W^*)\,[W^*E^*] - W^* \cdot E_{\rm tot}^*\right)/\partial W^* = 0 \tag{S.19}$$

$$\partial\left(k_{cat}^*[W^*E^*] - k_{cat}[WE]\right)/\partial W^* = 0 \tag{S.20}$$

$$\partial\left(I + f\,(W^*) - rE_{\rm tot}\right)/\partial W^* = 0 \tag{S.21}$$

$$\partial\left(W + W^* + [WE] + [W^*E^*] - W_{\rm tot}\right)/\partial W^* = 0 \tag{S.22}$$

After expansion, the equations become

$$(K_m + W)\,[WE] - W \cdot E_{\rm tot} = 0, \tag{S.23}$$

$$(K_m^* + W^*)\,[W^*E^*] - W^* \cdot E_{\rm tot}^* = 0, \tag{S.24}$$

$$k_{\rm cat}^*[W^*E^*] - k_{\rm cat}[WE] = 0, \tag{S.25}$$

$$I + f\,(W^*) - rE_{\rm tot} = 0, \tag{S.26}$$

$$W + W^* + [WE] + [W^*E^*] - W_{\rm tot} = 0, \tag{S.27}$$

$$[WE]\frac{\partial W}{\partial W^*} + (K_m + W)\,\frac{\partial [WE]}{\partial W^*} - E_{\rm tot}\frac{\partial W}{\partial W^*} - W\frac{\partial E_{\rm tot}}{\partial W^*} = 0, \tag{S.28}$$

$$[W^*E^*] + (K_m^* + W^*)\,\frac{\partial [W^*E^*]}{\partial W^*} - E_{\rm tot}^* = 0, \tag{S.29}$$

$$k_{\rm cat}^*\frac{\partial [W^*E^*]}{\partial W^*} - k_{\rm cat}\frac{\partial [WE]}{\partial W^*} = 0, \tag{S.30}$$

$$\frac{df\,(W^*)}{dW^*} - r\frac{\partial E_{\rm tot}}{\partial W^*} = 0, \tag{S.31}$$

$$\frac{\partial W}{\partial W^*} + 1 + \frac{\partial [WE]}{\partial W^*} + \frac{\partial [W^*E^*]}{\partial W^*} = 0. \tag{S.32}$$

By substituting the values of the parameters k_{on}, k_{off}, k_{cat}, k^*_{on}, k^*_{off}, k^*_{cat}, $F_{\max}$, $W_{0.5}$, n, r, W_{tot}, and E^*_{tot} into the above 10 equations, the solutions to the variables (I, W^*, W, $[WE]$, $[W^*E^*]$, E_{tot}, $\partial W/\partial W^*$, $\partial[WE]/\partial W^*$, $\partial[W^*E^*]/\partial W^*$, $\partial E_{\text{tot}}/\partial W^*$) are determined.

If all the solutions are meaningless, then singularities do not exist; and the response curve is a graded curve, not a bistable one. If meaningful solutions exist, then they must present as a pair: one is denoted by (I_{on}, W^*_{on}, W_{on}, $[WE]_{\text{on}}$, $\cdots$) and the other is denoted by (I_{off}, W^*_{off}, W_{off}, $[WE]_{\text{off}}$, $\cdots$). In this way, we have determined the two bifurcation points (I_{on}, W^*_{on}) and (I_{off}, W^*_{off}).

Stability analysis

The stability of any point of a response curve can be determined by calculating the eigenvalues of the Jacobian matrix associated with that point. The point is stable if and only if every eigenvalue has negative real part.

There are seven variables in the mathematical model. Because of the three constrains (Eqs. (8–10)), there are only four independent variables, whose time evolutions are described by

$$\frac{d\,[W^*]}{dt} = -k^*_{\text{on}}\,[W^*]\left([E^*_{\text{tot}}] - [W^*E^*]\right) + k^*_{\text{off}}\,[W^*E^*] + k_{\text{cat}}\,[WE]\,, \tag{S.33}$$

$$\frac{d\,[WE]}{dt} = k_{\text{on}}\left([W_{\text{tot}}] - [W^*] - [WE] - [W^*E^*]\right)\left([E_{\text{tot}}] - [WE]\right) - \left(k_{\text{off}} + k_{\text{cat}}\right)[WE]\,, \tag{S.34}$$

$$\frac{d\,[W^*E^*]}{dt} = k^*_{\text{on}}\,[W^*]\left([E^*_{\text{tot}}] - [W^*E^*]\right) - \left(k^*_{\text{off}} + k^*_{\text{cat}}\right)[W^*E^*]\,, \tag{S.35}$$

$$\frac{d\,[E_{\text{tot}}]}{dt} = I + f\left([W^*]\right) - r[E_{\text{tot}}]. \tag{S.36}$$

The associated Jacobian matrix is

$$\Phi = \begin{bmatrix} \Phi_{11} & \Phi_{12} & \Phi_{13} & \Phi_{14} \\ \Phi_{21} & \Phi_{22} & \Phi_{23} & \Phi_{24} \\ \Phi_{31} & \Phi_{32} & \Phi_{33} & \Phi_{34} \\ \Phi_{41} & \Phi_{42} & \Phi_{43} & \Phi_{44} \end{bmatrix}, \tag{S.37}$$

where

$\Phi_{11} = -k_{\text{on}}^{*}\left([E_{\text{tot}}^{*}] - [W^{*}E^{*}]\right)$, $\Phi_{12} = k_{\text{cat}}$,

$\Phi_{13} = k_{\text{off}}^{*} + k_{\text{on}}^{*}[W^{*}]$, $\Phi_{14} = 0$,

$\Phi_{21} = -k_{\text{on}}\left([E_{\text{tot}}] - [WE]\right)$, $\Phi_{22} = -k_{\text{off}} - k_{\text{cat}} + k_{\text{on}}([W^{*}] + [W^{*}E^{*}] - [W_{\text{tot}}] - [E_{\text{tot}}])$,

$\Phi_{23} = -k_{\text{on}}\left([E_{\text{tot}}] - [WE]\right)$, $\Phi_{24} = k_{\text{on}}([W_{\text{tot}}] - [W^{*}] - [WE] - [W^{*}E^{*}])$,

$\Phi_{31} = k_{\text{on}}^{*}\left([E_{\text{tot}}^{*}] - [W^{*}E^{*}]\right)$, $\Phi_{32} = 0$,

$\Phi_{33} = -k_{\text{on}}^{*}[W^{*}] - k_{\text{off}}^{*} - k_{\text{cat}}^{*}$, $\Phi_{34} = 0$,

$\Phi_{41} = df\left([W^{*}]\right)/d[W^{*}]$, $\Phi_{42} = 0$,

$\Phi_{43} = 0$, and $\Phi_{44} = -r$.

Notice the expression for Φ_{41}. If $f\left([W^{*}]\right)$ is a nonlinear feedback (Eq. (12)), then

$$\Phi_{41} = \frac{df\left([W^{*}]\right)}{d[W^{*}]} = \frac{F_{\max}W_{0.5}^{n}}{\left(W_{0.5}^{n} + [W^{*}]^{n}\right)^{2}}.$$

If $f\left([W^{*}]\right)$ is a linear feedback ($f\left([W^{*}]\right) = c[W^{*}]$), then

$$\Phi_{41} = \frac{df\left([W^{*}]\right)}{d[W^{*}]} = c.$$

Given a point of the response curve, one substitutes the state and parameter values into Eq. (S.37), resulting in a numerical 4×4 Jacobian matrix. One then obtains its eigenvalues by softwares such as Matlab or Mathematica.

By stability analysis, we found that the graded response curve is always stable. For the bistable response, the upper and lower branches are always stable; while the middle branch is always unstable.

Idealized model

We follow [1] by assuming that the substrate W_{tot} is in large excess over the enzymes, which allows us to drop $[WE] + [W^{*}E^{*}]$ in Eq. (8) and yields

$$W + W^{*} = W_{\text{tot}}. \tag{S.38}$$

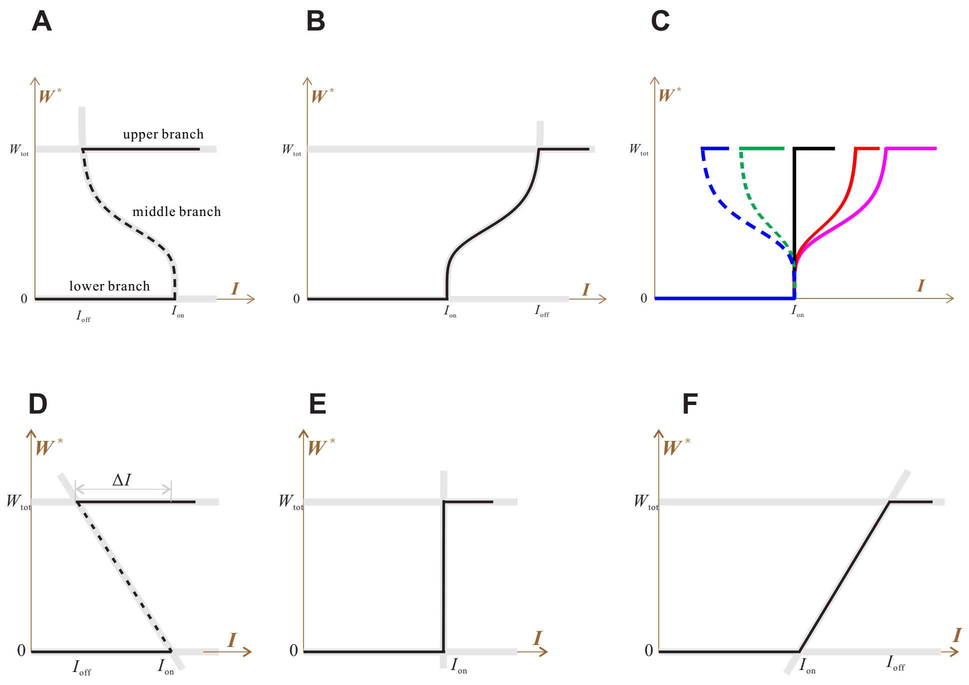

Figure S.2: **Response curves of RCM with feedback from W^* to $E_{\rm tot}$, obtained by the idealized model**. The upper panel is for the case when the feedback is the Hill function. (**A**) Positive feedback. (**B**) Negative feedback. (**C**) Combined illustration. The lower panel is for the case when the feedback is linear. (**D**) Positive feedback. (**E**) Null feedback. (**F**) Negative feedback.

With this simplification, the steady-state equations (S.1–S.10) reduces to a single equation

$$\frac{I+f\left(W^*\right)-rkE^*_{\rm tot}}{I}\left(\frac{W^*}{W_{\rm tot}}\right)^3-\frac{I+f\left(W^*\right)}{I}K^*\left(\frac{W^*}{W_{\rm tot}}\right)$$
$$+\left(\frac{I+f\left(W^*\right)}{I}\left(K^*-1\right)+\frac{rk\left(K+1\right)E^*_{\rm tot}}{I}\right)\left(\frac{W^*}{W_{\rm tot}}\right)^2=0, \qquad \text{(S.39)}$$

which is still complex and difficult to analyze.

Equation (S.39) can be reduced by using the limit condition $K\rightarrow 0$ and $K^*\rightarrow 0$, which parallels with the assumption of large $W_{\rm tot}$. Indeed, from Eq. (11) one sees that the largeness of $W_{\rm tot}$ implies the smallness of K and K^*. By this simplification, Eq. (S.39) is further reduced and can be factored into three terms

$$\left(W^*-0\right)\left(W^*-W_{\rm tot}\right)\left(I+f\left(W^*\right)-rkE^*_{\rm tot}\right)=0. \qquad \text{(S.40)}$$

Equation (S.40) explicitly tells the shape of the response curve (Fig. S.2*A*). The factors $W^*-0=$

0 and $W^* - W_{\text{tot}} = 0$ correspond to the lower and upper horizontal lines, respectively. The third factor, containing the Hill function $f(W^*)$, corresponds to a 'vertically placed' sigmoidal curve that intersects the lower and upper horizontal lines at $(I_{\text{on}}, 0)$ and $(I_{\text{off}}, W_{\text{tot}})$, respectively, where

$$\begin{aligned} I_{\text{on}} &= rkE^*_{\text{tot}}, \\ I_{\text{off}} &= rkE^*_{\text{tot}} - f(W_{\text{tot}}) . \end{aligned} \tag{S.41}$$

By defining $\Delta I = I_{\text{on}} - I_{\text{off}}$, we have

$$\begin{aligned} I_{\text{on}} &= rkE^*_{\text{tot}}, \\ \Delta I &= f(W_{\text{tot}}) . \end{aligned} \tag{S.42}$$

The above elucidation is also applicable when the feedback is linear (Fig. S.2*D*,*E*,*F*).

Note that this method of determining I_{on} and ΔI is much simpler than bifurcation analysis of the full model, which embodies the power of idealization. One should also be convinced of the validity of our idealization—the ideal response curve in Fig. S.2*A* is indeed the limit of response curves in Fig. S.1*A* (obtained by the full model). To further demonstrate the validity of idealization, we sample the space of response curves by using random parameter values. For each bistable curve, we compare I_{on} and ΔI obtained by Eq. (S.42) of the idealized model with those obtained by bifurcation analysis of the full model. Figures 2*A*, 2*B* illustrate the results. The validity of idealization extends to the case when the feedback is double negative (Fig. 2*D*,*E*).

RCM with other feedbacks: a summary

We have analyzed RCM with positive feedback $W^* \to E_{\text{tot}}$ in detail. For the other feedbacks, the mathematical model is the same except the part describing regulations to E_{tot} or E^*_{tot} (the second column of Fig. S.3). The third column of Fig. S.3 lists results obtained from the idealized model.

	Feedbacks	Enzyme regulation	Idealized model results
**1**	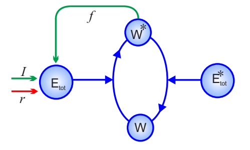	$\frac{dE_{tot}}{dt} = I + f(W^*) - rE_{tot}$ $E^*_{tot} = \text{const}$	$(W^* - 0)(W^* - W_{tot})$ $(I + f(W^*) - rkE^*_{tot}) = 0$ $I_{on} = rkE^*_{tot}$ $\Delta I = f(W_{tot})$
**2**	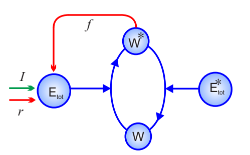	$\frac{dE_{tot}}{dt} = I - rE_{tot} - f(W^*)E_{tot}$ $E^*_{tot} = \text{const}$	$(W^* - 0)(W^* - W_{tot})$ $(I - (r + f(W^*))kE^*_{tot}) = 0$ $I_{on} = rkE^*_{tot}$ $\Delta I = -f(W_{tot})kE^*_{tot}$
**3**	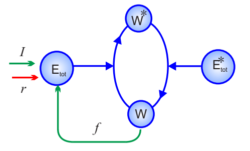	$\frac{dE_{tot}}{dt} = I + f(W) - rE_{tot}$ $E^*_{tot} = \text{const}$	$(W^* - 0)(W^* - W_{tot})$ $(I + f(W_{tot} - W^*) - rkE^*_{tot}) = 0$ $I_{off} = rkE^*_{tot}$ $\Delta I = -f(W_{tot})$
**4**	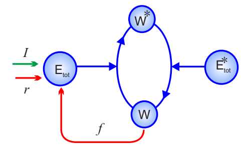	$\frac{dE_{tot}}{dt} = I - rE_{tot} - f(W)E_{tot}$ $E^*_{tot} = \text{const}$	$(W^* - 0)(W^* - W_{tot})$ $(I - (r + f(W_{tot} - W^*))kE^*_{tot}) = 0$ $I_{off} = rkE^*_{tot}$ $\Delta I = f(W_{tot})kE^*_{tot}$
**5**	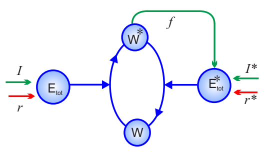	$\frac{dE_{tot}}{dt} = I - rE_{tot}$ $\frac{dE^*_{tot}}{dt} = I^* + f(W^*) - r^*E^*_{tot}$	$(W^* - 0)(W^* - W_{tot})$ $(I - (I^* + f(W^*))rk/r^*) = 0$ $I_{on} = I^*rk/r^*$ $\Delta I = -f(W_{tot})rk/r^*$
**6**	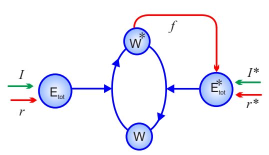	$\frac{dE_{tot}}{dt} = I - rE_{tot}$ $\frac{dE^*_{tot}}{dt} = I^* - r^*E^*_{tot} - f(W^*)E^*_{tot}$	$(W^* - 0)(W^* - W_{tot})$ $(Ir^* + If(W^*) - rkI^*) = 0$ $I_{on} = I^*rk/r^*$ $\Delta I = \frac{f(W_{tot})I^*rk}{r^*(r^* + f(W_{tot}))}$
**7**	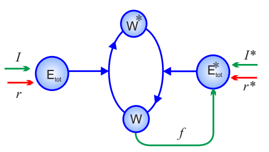	$\frac{dE_{tot}}{dt} = I - rE_{tot}$ $\frac{dE^*_{tot}}{dt} = I^* + f(W) - r^*E^*_{tot}$	$(W^* - 0)(W^* - W_{tot})$ $(I - (I^* + f(W_{tot} - W^*))rk/r^*) = 0$ $I_{off} = I^*rk/r^*$ $\Delta I = f(W_{tot})rk/r^*$
**8**	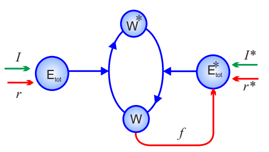	$\frac{dE_{tot}}{dt} = I - rE_{tot}$ $\frac{dE^*_{tot}}{dt} = I^* - r^*E^*_{tot} - f(W)E^*_{tot}$	$(W^* - 0)(W^* - W_{tot})$ $(Ir^* + If(W_{tot} - W^*) - rI^*k^*_{cat}/k_{cat}) = 0$ $I_{off} = I^*rk^*_{cat}/(r^*k_{cat})$ $\Delta I = \frac{-f(W_{tot})I^*rk^*_{cat}}{r^*k_{cat}(r^* + f(W_{tot}))}$
	**A**	**B**	**C**

Figure S.3: **A summary of regulated RCM models.**

Insights into development: examples

In the main text, we mentioned that exclusive differentiation is sometimes necessary. A case in point is the RCM of Yan during the development of the *Drosophila* trachea, a ramified network of epithelial tubes formed through a successive branching events [24]. Figure S.4*A* illustrates a tracheal primary branch, consisting of cells of the same type originally. These cells migrate towards some nearby mesenchymal tissue that secretes the attractant Bnl. In the following, our elucidation is in terms of the left hemisegment of the primary branch. The top cell (colored in magenta) differentiates into a new cell type, from which a new tracheal branch will sprout out. To prevent excessive branching, the trailing cell is not allowed to differentiate.

How to specify disparate fates for two identical cells so close in the space? One may argue that the top cell is a little closer to the Bnl source. But the trailing cell migrates towards the Bnl source with the same speed and it should have equal chance of activation. The question is answered if the trailing cell's I_{on} is markedly raised. Indeed, the trailing cell's MAPK is inhibited by Notch signalling [25, 26], which is not a feedback from the cell's own Yan activity. In fact, it is the top cell's MAPK that inhibits the trailing cell's MAPK (through Notch/Delta signalling; see Fig. S.4*B*). This lateral inhibition, as a nonfeedback regulation, can raise the trailing cell's I_{on}. The resultant delay might be sufficient to deprive the cell's differentiation opportunity (Fig. S.4*C*).

The above example is about specifying two cell fates by a morphogen gradient. In fact, a single morphogen gradient can even specify three or more cell fates [23, 31], by activating many genes. If these genes' I_{on} values are the same, then only two cell fates will result (similar to the example in the main text). To specify more cell fates, these I_{on} values must be well separated so that the on/off combinations are large enough to generate differences. Several mechanisms exist to separate the I_{on} values, e.g., cell-cell communication (Notch/Delta signalling) mentioned in the main text. Alternatively, I_{on}-tuning can be realized intracellularly through mutual inhibitions among the genes. Figure S.5 illustrates three genes X, Y, and Z whose on/off combinations determine four cell types. Through mutual inhibitions among the three genes, their I_{on} values are well separated, which allows for the generation of four cell types by a single morphogen gradient.

Note that decoupling of sensitivity tuning is crucial to the success of the I_{on}-separation strategy.

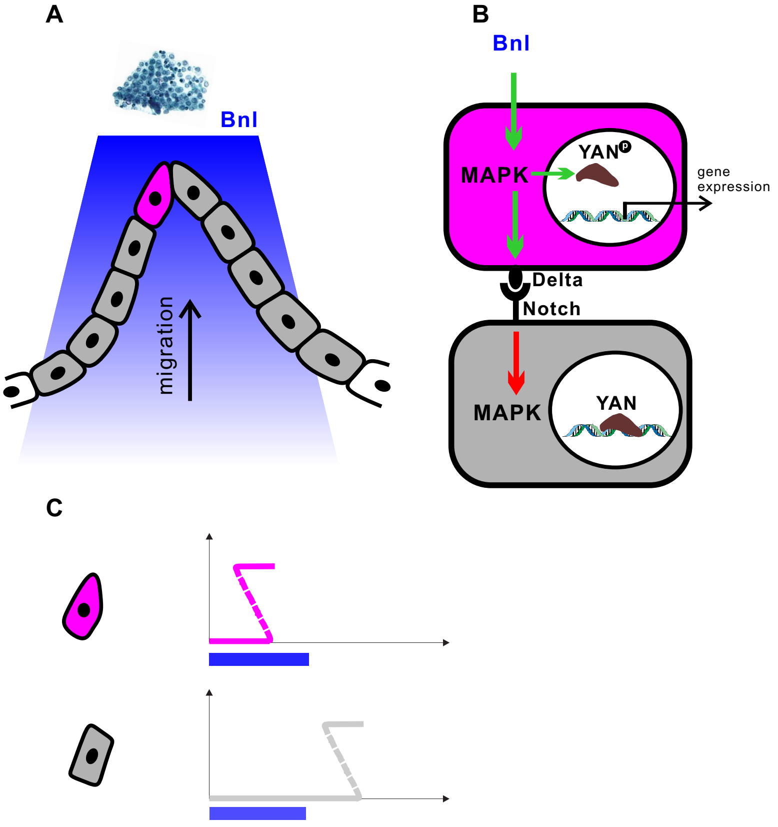

Figure S.4: **Early development of drosophila trachea system**. (**A**) A primary branch migrates and differentiates in response to a gradient of Bnl secreted by nearby mesenchymal tissue. (**B**) Notch/Delta lateral inhibition from the top cell to the trailing cell. The consequence is inhibition of Yan phosphorylation in the trailing cell due to the raised I_{on}. (**C**) The two response curves are well separated due to Notch/Delta lateral inhibition. As a consequence, only the top cell is activated, although the trailing cell's local Bnl concentration is only slightly smaller than that of the top cell.

If crosstalks are severe, a nonfeedback inhibition delays the response (desired) but at the same time deforms the response curve (undesired; for the activation quality becomes degraded), leading to inadequate gene expressions and thus abnormal cell types.

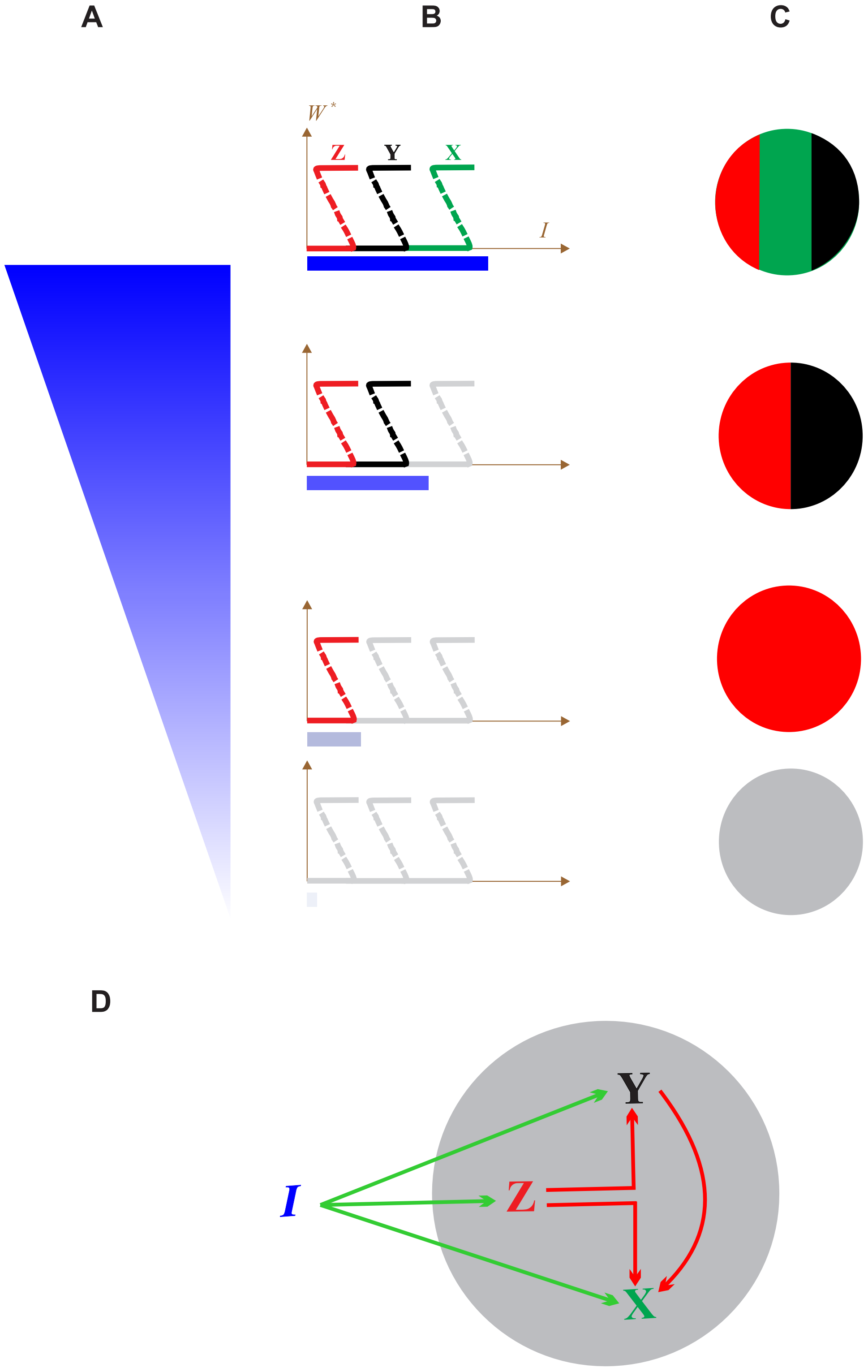

Figure S.5: **Specification of four cell types by a single morphogen gradient**. (**A**) The morphogen concentration decreases from top down. (**B**) Response curves of four cells at different positions along the gradient. The responses are different among the four cells, due to the well-separated gene activation thresholds. (**C**) The generation of four cell types. From top down, the cells have 3, 2, 1, 0 genes activated. (**D**) To separate the thresholds, gene Z inhibits both genes X and Y, while gene Y inhibits gene X.
